# Cerebral cortical hemodynamic metrics to aid in assessing pain levels? A pilot study of functional near-infrared spectroscopy

**DOI:** 10.3389/fnins.2023.1136820

**Published:** 2023-03-15

**Authors:** Jiahao Du, Ping Shi, Fanfu Fang, Hongliu Yu

**Affiliations:** ^1^Institute of Rehabilitation Engineering and Technology, University of Shanghai for Science and Technology, Shanghai, China; ^2^Department of Rehabilitation Medicine, Changhai Hospital, Naval Medical University, Shanghai, China

**Keywords:** blood volume dynamics, functional near-infrared spectroscopy, pain tests, wrist-ankle acupuncture, cervical-shoulder syndrome

## Abstract

**Introduction:**

Establishing an accurate way to quantify pain is one of the most formidable tasks in neuroscience and medical practice. Functional near-infrared spectroscopy (fNIRS) can be utilized to detect the brain’s reaction to pain. The study sought to assess the neural mechanisms of the wrist-ankle acupuncture transcutaneous electrical nerve stimulation analgesic bracelet (*E-WAA*) in providing pain relief and altering cerebral blood volume dynamics, and to ascertain the reliability of cortical activation patterns as a means of objectively measuring pain.

**Methods:**

The participants (mean age 36.6 ± 7.2 years) with the cervical-shoulder syndrome (CSS) underwent pain testing prior to, 1 min following, and 30 min after the left point Jianyu treatment. The *E-WAA* was used to administer an electrical stimulation therapy that lasted for 5 min. A 24-channel fNIRS system was utilized to monitor brain oxyhemoglobin (HbO) levels, and changes in HbO concentrations, cortical activation areas, and subjective pain assessment scales were documented.

**Results:**

We discovered that HbO concentrations in the prefrontal cortex significantly increased when CSS patients were exposed to painful stimuli at the cerebral cortex level. The second pain test saw a considerable decrease in the average HbO change amount in the prefrontal cortex when *E-WAA* was applied, which in turn led to a reduction in the amount of activation and the size of the activated area in the cortex.

**Discussion:**

This study revealed that the frontal polar (FP) and dorsolateral prefrontal cortex (DLPFC) were linked to the analgesic modulation activated by the *E-WAA*.

## 1. Introduction

Pain, according to the International Association for the Study of Pain, is “An unpleasant sensory and emotional experience associated with, or resembling that associated with, actual or potential tissue damage” (Raja et al., 2020). Developing an accurate and quantifiable way to measure pain is one of the most significant issues in neuroscience and clinical medicine. The reliability of current pain assessment strategies, which primarily rely on patient-reported information and medical evaluations, is questionable when applied to different types and sources of pain. In addition, the variability of pain scores and the difficulty of obtaining accurate patient feedback may conceal the severity of their condition, resulting in inadequate treatment for those affected, including those affected by stroke or dementia, as well as infants. The scientific credibility of clinical analgesic interventions is drastically reduced by this phenomenon.

Recently, due to researchers gaining better comprehension of pain’s causes and the brain’s associated neural pathways, as well as the emergence of more sophisticated neuroimaging methods, it has been established that a quantitative assessment of brain nociception in living organisms is possible (Ploner et al., 2017). The various techniques of functional neuroimaging, such as functional near-infrared spectroscopy (fNIRS) (Rojas et al., 2017), functional magnetic resonance imaging (fMRI) (Wager et al., 2013), and electroencephalogram (EEG), as shown in Table 1, are widely utilized. fMRI is the most reliable method of monitoring brain activity related to pain, however, the use of this technique necessitates physical limitations on the head and body, making it impractical for use in actual clinical settings. In comparison, fNIRS is capable of monitoring local cerebral blood flow by detecting the alteration of near-infrared light wavelengths between 650 and 1,000 nm (Ferrari and Quaresima, 2012), and it is equipped with a high temporal resolution (Mehta and Parasuraman, 2013), motion compatibility (Scarapicchia et al., 2017), portability, and the ability to detect pain in actual time in intricate clinical contexts (Basura et al., 2018).

**TABLE 1:** Comparison of mainstream functional brain imaging techniques.

	fMRI	EEG	fNIRS
Detection parameters	BOLD signal	Neural activity	HbO
Detection depth	Superficial and deep cortex	Superficial cortex	Superficial cortex
Spatial resolution/cm	High	Low	Low
Temporal resolution/Hz	Low	High	High
Anti-motion interference	Low	Low	High
Anti-electromagnetic interference	–	Low	High
Application environment	Large, specialized instrument room and posture limitation	Not suitable for electromagnetic interference environment	No restrictions
Mobility	–	Low	High

Research has indicated that fNIRS is a viable option for observing cortical hemodynamic changes in response to both experimentally and clinically induced pain in humans (Barati et al., 2013; Karunakaran et al., 2021). Studies involving pain have utilized fNIRS to measure the brain activity of newborns (Viola et al., 2014), healthy adults (Rojas et al., 2017; Han et al., 2018), and those with chronic pain (Xu et al., 2021) in response to unpleasant pain/stimuli. Studies have demonstrated that the introduction of unpleasant stimuli/pain to both healthy individuals and those with headaches is associated with an increase in prefrontal cortical activity (Brighina et al., 2011; Berger et al., 2019). Contrastingly, experiments on patients experiencing pain after a dental extraction (Derbyshire et al., 1999), people suffering from rheumatoid arthritis (Sandström et al., 2019), and even healthy participants (Sava et al., 2014) all demonstrated that painful stimulation causes a reduction in prefrontal cortical activity. The exact mechanism behind the prefrontal cortical activity caused by pain remains obscure, potentially being linked to the area of the body and the kind of pain experienced. A sizeable proportion of Chinese people suffer from cervical-shoulder syndrome (CSS), estimated to be between 8.1 and 19.1% (Lv et al., 2018), and this group was chosen as the sample for this research (Cai et al., 2016). Patients with CSS typically receive pharmacological and physical factor treatments in a clinical setting, which have been found to be effective in relieving their symptoms. Recently, a new method to wrist-ankle acupuncture-based electrical stimulation analgesic bracelet *E-WAA* has been made available, and is more popular among CSS patients due to its non-invasiveness, convenience, and effectiveness. The efficacy of medications such as morphine in relieving pain has been validated through objective techniques such as fMRI and fNIRS (Peng et al., 2018c). Despite this, the effectiveness of *E-WAA* has been demonstrated in several studies (Yang et al., 2019; Song et al., 2021; Du et al., 2022), its analgesic mechanism remains to be explored.

This study used fNIRS to assess the impact of *E-WAA* on cerebral blood flow and cortical activation patterns. We observed the fNIRS signals originating from the prefrontal area, which is an important part of pain perception, and examined how *E-WAA* impacted brain activity in our study. We delved deeper to determine if these hemodynamic shifts could be employed as a marker to measure the intensity of pain in CSS patients, thereby amplifying our knowledge of the hemodynamic answer to nociception in the prefrontal cortex of CSS patients.

## 2. Materials and methods

### 2.1. Participants

Participants were recruited from a representative sample of teachers and students affiliated with the University of Shanghai for Science and Technology, all of whom were members of a society of different ages. Previous studies have suggested that sex-related hormones may confound the relationship between pain and analgesic response (Fillingim and Ness, 2000; Mogil, 2020). Therefore, only males were selected as participants in the present study. All participants were right-handed to avoid changes in functional responses due to functional lateralization of the brain. We enlisted 20 male participants with CSS, with an average age of 36.6 years and a range of 7.2 years, who agreed to participate in the study after being informed of its details. All procedures were approved by the ChiRCT ethics committee.

### 2.2. Experimental devices

**FIGURE 1:**
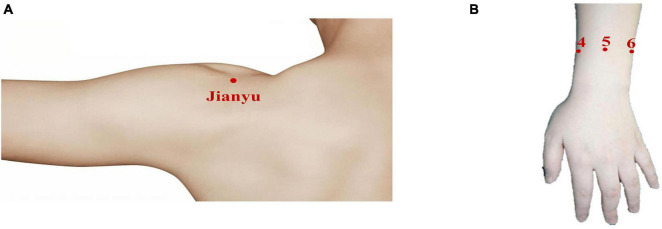
Schematic diagram of the positions of the experimental devices. **(A)** The pressure pain model—point Jianyu. Point Jianyu is situated on the front side of the shoulder blade, in the dip between the shoulder blade and the large protrusion of the arm bone (Tao et al., 2005). **(B)** The treatment site—the upper 5 area of the wrist. 4, 5, and 6 correspond to the upper 4 area, the upper 5 area, and the upper 6 area, respectively. The upper 5 area is located in the middle of the palm surface between the two most prominent long palmar tendons and the radial carpal flexor tendon.

The point Jianyu was the most common pressure point in CSS patients, shown in Figure 1A, so we used the pressure pain at the point Jianyu as the pain model for this experiment (Amiri et al., 2021; Uysal et al., 2022). The degree of muscular tissue hardness is an objective indicator of the change in pain, and according to Chinese medicine theory, the higher the value of the acupoint tissue, the greater the degree of pain (Chen et al., 2007; Finocchietti et al., 2011). The criteria for pressure pain were that the same force (3–4 kg/cm^2^) was applied to the patient to assess the patient’s subjective visual analog scale (VAS) score, objective tissue stiffness, and objective cerebral blood flow changes under the same force. A rapid muscle measurement device (OE-220: Ito Corporation, Tokyo, Japan) was used to record the change in tissue stiffness at the participant’s pain site before and after analgesia to reflect the change in pain.

Analgesic treatment is performed using a transcutaneous electrical nerve stimulation analgesic bracelet based on Chinese medicine wrist and ankle acupuncture (*E-WAA*) (Shi et al., 2020). It is a small portable non-invasive electrical stimulation device that can be worn on the wrist or ankle with Velcro to relieve or even treat pain in the human body, and its subjective analgesic effect has been demonstrated in several studies (Song et al., 2021; Du et al., 2022; Shi et al., 2022). The treatment site chosen for this experiment was the upper 5 area on the left hand, identified by the mechanism of WAA in traditional Chinese medicine (TCM) (Figure 1B). The upper 5 area is located in the middle of the palm surface between the two most prominent long palmar tendons and the radial carpal flexor tendon (Zhu et al., 2014). Due to the specificity of pain perception, the treatment parameters were self-adjusted: 0–100 V, 0–100 Hz.

To measure the changes in cerebral blood volume dynamics, we employed a continuous wave 24-channel fNIRS system (OXYMON MKIII; Artinis Co., Netherlands) using two wavelengths of near-infrared light (752 and 841 nm), as well as 24-channel probes (light sources and receivers). The distance between the light source and the receiver was 3 cm.

### 2.3. Experimental procedure

The study was conducted in an undisturbed laboratory where all lights were turned off (light intensity controlled below 100 lx). During the experiment, participants were expected to sit and remain as still as possible. The experimental procedures were performed by experienced traditional Chinese medical specialists.

**FIGURE 2:**
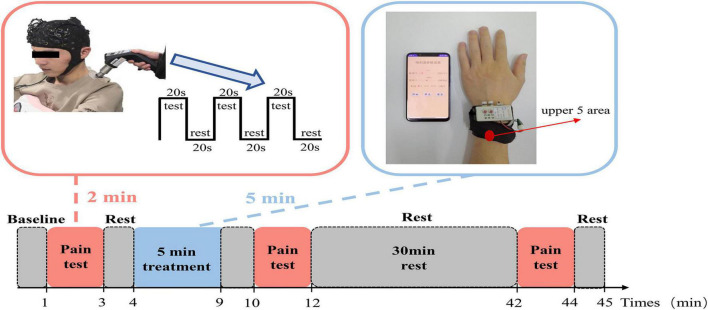
Experimental procedures.

The experimental procedure consisted of the first pain test-5 min of treatment-the second pain test-30 min of rest- the third pain test, with 1-minute rest intervals between blocks, as shown in Figure 2. The pain test consisted of 3 sets of 20 s of pressure pain and 20 s of rest. During the pain test, participants were simultaneously asked to score on a VAS. During the analgesic treatment, two output gold fingers of the *E-WAA* were placed on the participant’s left hand in the upper 5 area for 5 min.

### 2.4. fNIRS and measurement items

**FIGURE 3:**
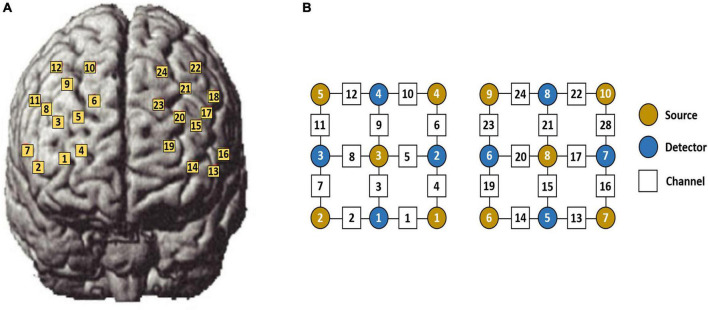
Functional near-infrared spectroscopy (fNIRS) photopole arrangement template. **(A)** Three-dimensional diagram of the distribution of 18 photopoles and 24 channels on the scalp surface. **(B)** Planar diagram of the photopolar template.

Functional near-infrared spectroscopy calculates the variation of oxyhemoglobin (HbO) (Berretz et al., 2021) and deoxyhemoglobin concentrations according to the modified Beer–Lambert law (Hoshi, 2003). In the present experiment, HbO concentrations were used for analysis only because of their high signal-to-noise ratio (Lacerenza et al., 2020). Since the fNIRS signal acquired in this experiment was obtained by measuring through a head-mounted photopolar array cap consisting of 12 light sources and 8 detectors, the spatial position of each measurement channel was first calibrated during the analysis of the data. Five participants were first randomly selected as the base template for the fNIRS channel position alignment and used to estimate the prefrontal cortical area covered by the detectors. When the fNIRS photopolar array was placed on the participant’s forehead, the positions of three cranial reference coordinates, namely the occipital ridge, the nasal root point, and the cranial capsule, were measured to translate the true stereotactic coordinates of the photopoles into the Montreal Neurological Institute (MNI) standard hemispheric regional coordinates used (Wagner et al., 2022). The locations of the 24 channels on the brain are shown in Figure 3.

The initially acquired fNIRS data had more noise and interference and required further processing. The noise interference of the acquired cerebral blood oxygen signal mainly comes from (1) the Global drift of the baseline hemodynamic signal due to physiological activities such as heart rate (0.8–2.0 Hz) and respiration (0.13–0.33 Hz). (2) Spontaneous neural activities in the brain such as low-frequency oscillations (LFO, e.g., Mayer wave, 0.1 Hz) and ultra-low frequency oscillations (VLFO, 0.03 Hz). (3) Motion artifact interference. In this paper, wavelet analysis with higher frequency was used for noise and interference processing. Specifically, this study uses the hemodynamic response function (HRF) and wavelet-minimum description length algorithm (wavelet-MDL) to process the acquired fNIRS signal is processed (Jang et al., 2009; Tak et al., 2011) to remove low-frequency noise and motion artifact noise. Baseline fitting by least squares removes the baseline drift that may be caused by the device itself. We analyzed the hemodynamic response associated with the timing of painful stimuli to verify brain activation.

We performed spatial alignment of the acquired spatial data using NIRS_SPM (Ye et al., 2009), an open-source toolkit developed by the Korea Institute of Science and Technology based on Matlab scripts. Based on a probabilistic alignment algorithm, the channel coordinates of each participant were aligned to the MNI standard template space. The average MNI coordinates for the whole group were then calculated, and the spatial alignment results are shown in Table 2, including the MNI coordinates for each channel, as well as the corresponding neuroanatomical labeling (AAL) and Brodmann functional partitioning at the maximum probability for each channel. In this experiment, the frontal polar (FP) region and the dorsolateral prefrontal cortex (DLPFC) of the prefrontal cortex were the regions of interest (ROIs).

**TABLE 2:** The cortical locations corresponding to the channels.

Channels	MNI coordinates	BA	AAL	Channels	MNI coordinates	BA	AAL
1	(36, 65, 8)	10	Frontal_Sup_R-FP	13	(−51, 46, 3)	46	Frontal_Mid_R-DLPFC
2	(52, 48, 5)	46	Frontal_Mid_R-DLPFC	14	(−38, 63, 3)	10	Frontal_Sup_L-FP
3	(40, 55, 26)	46	Frontal_Mid_R-DLPFC	15	(−40, 54, 23)	46	Frontal_Mid_L-DLPFC
4	(26, 70, 13)	10	Frontal_Sup_R-FP	16	(−56, 33, 10)	45	Frontal_Inf_Tri_Lpars triangularis Broca’s area
5	(29, 60, 29)	46	Frontal_Mid_R-DLPFC	17	(−46, 39, 30)	46	Frontal_Mid_R_R-DLPFC
6	(18, 60, 36)	9	Frontal_Sup_R-DLPFC	18	(−51, 26, 39)	46	Frontal_Mid_R_R-DLPFC
7	(58, 34, 12)	45	Frontal_Inf_Tri_Rpars triangularis Broca’s area	19	(−25, 69, 13)	10	Frontal_Sup_L-FP
8	(47, 41, 32)	46	Frontal_Mid_R_R-DLPFC	20	(−29, 57, 28)	46	Frontal_Mid_L-DLPFC
9	(35, 42, 45)	9	Frontal_Mid_R-DLPFC	21	(−34, 42, 41)	9	Frontal_Mid_L-DLPFC
10	(22, 43, 51)	9	Frontal_Sup_R-DLPFC	22	(−40, 25, 52)	9	Frontal_Mid_L-DLPFC
11	(54, 24, 37)	46	Frontal_Mid_R_R-DLPFC	23	(−18, 61, 34)	9	Frontal_Sup_L-DLPFC
12	(41, 25, 53)	9	Frontal_Mid_R-DLPFC	24	(−21, 44, 50)	9	Frontal_Sup_L-DLPFC

### 2.5. Statistical analysis

SPSS 21.0 statistical software was used for data analysis of VAS and tissue stiffness. The Shapiro–Wilk test was used to assess the normal distribution of the data. One-way repeated measures ANOVA with Bonferroni *post-hoc* test was performed on VAS and tissue stiffness parameters (expressed as mean ± standard deviation) for three pain tests that obeyed normal distribution. *P* < 0.05 was considered a statistically significant difference.

In addition, we used the statistical parameter mapping NIRS-SPM (SPM 8) tool in NIRS-lab 2017.6 and the Shapiro–Wilk test to analyze and verify the normality of the fNIRS data. To quantify the prefrontal cortical hemodynamic response to pain tests, a general linear model (GLM) was first fitted to the fNIRS data, and then the values obtained from the GLM for pain stimulus intervals and rest intervals were interpolated and smoothed over the general human brain. The expected Eulerian feature method based on Lipschitz-Killing curvature was used to control for group errors. The degree of activation of each channel was expressed as a regression coefficient (β), and Bonferroni correction was then used for multiple comparisons. The differences between the values were ascertained by conducting an independent sample *t*-test, and then a one-way ANOVA was used to compare the groups, with a significance criterion of *P* < 0.05 for all analyses.

## 3. Results

The data in Table 3 reveals that there was a significant change in VAS and tissue stiffness between the three pain assessments, displaying a substantial divergence in VAS and tissue stiffness before and after treatment (*F* = 0.041, *P* < 0.001; *F* = 3.619, *P* = 0.042). The *post-hoc* tests showed that the differences between pre-treatment and 1-minute post-treatment, as well as 30 min post-treatment, were statistically significant, with *P* < 0.05. And the results of the tests between 1 and 30 min post-treatment had a *P*-value of 1.00, which is higher than 0.05, suggesting that there was no substantial variation. The present results confirm that we induced analgesia, as known from previous studies (Shi et al., 2022), *E-WAA* was able to significantly reduce participants’ VAS and tissue stiffness.

**TABLE 3:** Comparison of VAS and tissue stiffness for three pain tests.

Indicator	*n*	The first pain test	The second pain test	The third pain test	*F*	*P*
VAS	20	5.33 ± 1.41	4.00 ± 1.12	3.78 ± 1.30	14.041	<0.001*
Tissue stiffness/%	20	57.63 ± 18.75	48.25 ± 8.49	47.67 ± 14.35	3.619	0.042*

*P < 0.05.

**FIGURE 4:**
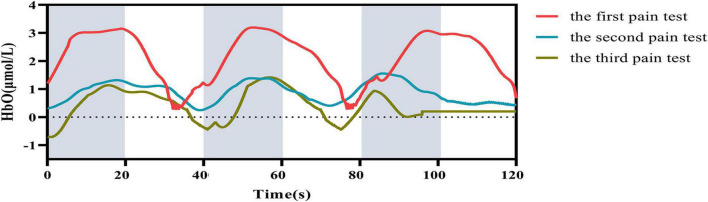
Time series of changes in mean HbO concentrations for three pain tests. The solid line indicates the average of the concentrations, red indicates the first pain test, blue indicates the second pain test, and green indicates the third pain test. The shaded part indicates the pain stimulation task, which lasted 20 s.

A total of 24 channels of fNIRS signals were acquired, 4 of which were in the FP–Ch1, Ch4, Ch14, and Ch19–and the remaining 18 channels were situated in the DLPFC- Ch2, Ch3, Ch5, Ch6, Ch8–Ch13, Ch15, Ch17, Ch18, and Ch20–Ch24, respectively. Figure 4 illustrates the alterations in the average HbO concentration of the ROI-associated channels of the participants over the three pain tests. The graph indicates that the average HbO concentration had an impressive recurrent pattern over the three pain trials. When participants received a pain stimulus (represented by gray shading in the graph) the ROIs were activated, which was evidenced by a steadily increasing graph culminating in a peak. Following treatment with *E-WAA*, the average central HbO concentration in the second pain test (red curve) was notably lower than that of the initial pain test (blue curve) after 1 min. Following 30 min of rest, the average central change in HbO concentration for the third pain test (green curve) was comparable to the preceding two pain tests.

**FIGURE 5:**
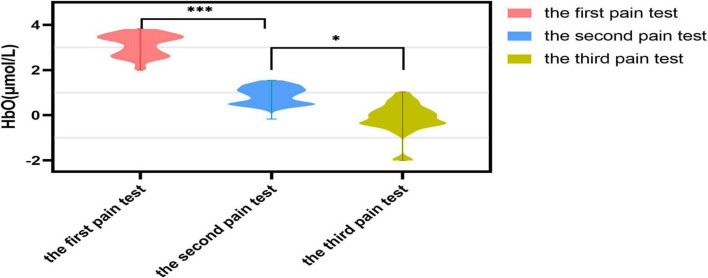
Differences in HbO levels between the three pain tests. *P < 0.05, ***P < 0.001.

Moreover, Figure 5 illustrates the contrast in the mean HbO concentrations for the ROI when subjected to the three pain tests. The ANOVA results revealed a notable reduction in mean HbO levels for participants in the second pain test when compared to the first one, which was statistically significant (*P* = 0.001). Despite this, the decline in HbO levels remained statistically significant when the third pain test was conducted compared to the second pain test (*P* = 0.034).

**FIGURE 6:**
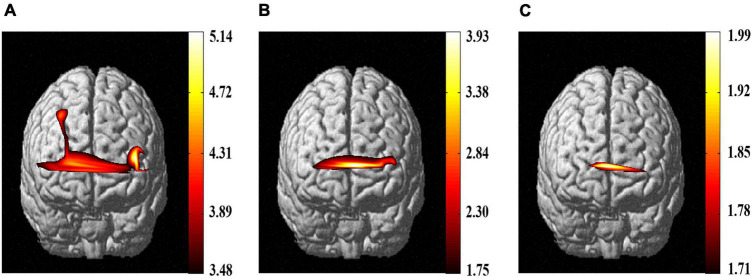
Outcomes of brain activity in response to three pain tests. **(A)** The first pain test. **(B)** The second pain test. **(C)** The third pain test. The colored bars on the right graph indicate *t*-values.

The prefrontal region of the brain is the primary focus of Figure 6, which shows the cortical activation outcomes of the participants’ ROIs for the three pain tests. The findings revealed that during the three pain experiments, the participants’ cerebral cortex exhibited a variety of activation degrees and ranges, distinguished by colors representing the intensity of the neural activation, from dark red to light yellow, signifying a progressive increase in activation. The initial experiment indicated that the FP and DLPFC areas of the brain were highly stimulated when the participants experienced pain in their point Jianyu (as seen in Figure 6A). Following electrical stimulation treatment, a second pain test was conducted 5 min later, which showed a considerable decrease in FP activation amongst the participants, whereas DLPFC had almost no detectable activation (Figure 6B). Upon the administration of the third stimulus, a limited region of FP was triggered within the prefrontal cortex of the individuals (Figure 6C). The results of the *E-WAA* treatment, combined with a period of recuperation, suggested that the participants’ ROI was less responsive to painful stimuli, resulting in generally decreased activation levels.

**FIGURE 7:**
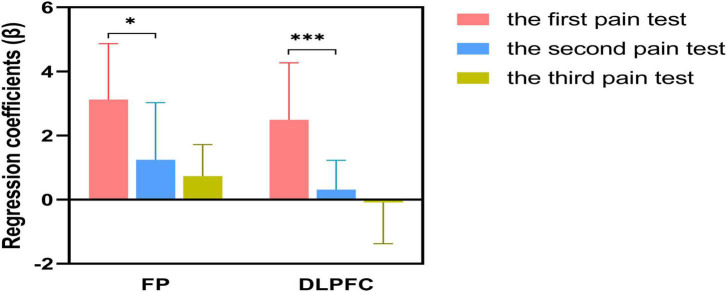
The β values of the regions of interest (ROI) were extracted from the results of the three pain tests. *P < 0.05, ***P < 0.001.

In addition, Figure 7 displays the regression coefficients (β) for the 3 experimental conditions at the 2 ROIs, which were analyzed statistically. The β values of both ROI regions were consecutively lower for all three pain tests and the first two tests created considerable disparities in the stimulation intensities of the FP and DLPFC regions (FP: *P* < 0.05; DLPFC: *P* < 0.001).

## 4. Discussion

Most pain assessment methods are based solely on patient-reported responses, which clearly renders them subjective rather than objective. In this experiment, an OE-220 tissue hardness meter is employed to measure the alteration in muscle firmness in numerical terms, thus allowing for a relatively impartial evaluation of the impact of pain on muscle tension. The OE-220 takes the mean of three measurements to compensate for any potential momentary discrepancies in the readings. The data in Table 3 showing the single factor variance of VAS and tissue stiffness confirm previous findings that electrical stimulation therapy can provide immediate and efficient pain relief. Through the activation of nerve endings, electrical stimulation can induce a form of pain relief that is not dependent on its physical location. A pain patient’s lesion site has a heightened sensory threshold, making them more sensitive to mild stimuli like electrical stimulation, which can lead to beneficial results such as reduced muscle spasms, improved circulation, and decreased pain when compared to a healthy individual. Conversely, the gate control theory of pain states that transcutaneous electrical nerve stimulation (TENS) stimulation leads to the activation of wide-diameter Aβ fibers and the inhibition of the painful feelings of the skin area nearby, which are generated by small-diameter, slow-conducting Aα and C fibers in the dorsal horn.

This research investigated the hemodynamic effects on the prefrontal cortex associated with the utilization of TENS analgesia as a means of furthering exploration into neural modulation. The prefrontal cortex is mainly connected to how the body and mind respond to pain. Various methods of alleviating discomfort have been identified, such as repetitive transcranial magnetic stimulation, transcranial direct current stimulation, antidepressant medication, acupuncture, cognitive-behavioral therapy, mindfulness, music, exercise, assistance from a partner, empathy, meditation, and prayer (Ren et al., 2017). Previous research has elucidated the part that the prefrontal cortex plays in placebo analgesia, as well as the relationship between pain and depression, anxiety, and cognitive decline (Peng et al., 2018b). This experimental study examined the FP and DLPFC, two key regions of the prefrontal cortex. The results of Figures 4, 5 demonstrate that CSS sufferers were responsive to painful stimuli, as their HbO levels noticeably changed when subjected to pain. The HbO level of the FP and DLPFC rose drastically in response to the pain stimulus, yet their overall center levels decreased significantly after the three pain tests. The data from this experiment suggest that the pain stimulus caused an increased level of HbO in the FP and DLPFC, indicating that these areas were activated. This study demonstrated a decrease in VAS scores and muscle tissue hardness, which is in agreement with the previous research finding that the DLPFC is associated with a reduction in secondary hyperalgesia.

The brain imaging results in Figure 6 of this study provided a visual representation of the cortical activation level of ROIs in the pain test, and a marked difference was observed when *E-WAA* was administered to the participants. The *t*-value map demonstrated a marked contrast between the task and the other conditions, implying that the hemodynamic response of the participants altered during the experiment, and with the implementation of *E-WAA*, the participants were less responsive to the painful stimulus. After considering the limitations of the t-map in displaying the spatial activity of the relevant areas during the task, further examination of the channel-directional hemodynamic response was conducted to evaluate the exact activity of FP and DLPFC; this is represented in Figure 7, which shows the mean activation level of both ROIs. The neural functional system’s FP, which is responsible for self-referencing, attention regulation, working memory, decision-making, and salience detection (Peng et al., 2018a), has a vital cognitive function in handling pain, such as being able to properly direct attention, recognize pain and respond to it. Studies involving fNIRS have indicated that FP is active in the presence of experimental pain and clinical pain, as well as in response to analgesics. It is thought that the DLPFC, similar to the FP, plays a role in more complex cognitive processes and information processing, such as attending to tasks, maintaining information in mind, and inhibiting inappropriate responses (Seminowicz and Moayedi, 2017). Studies using fNIRS have demonstrated that the DLPFC is particularly active during experimental pain, and has been observed to be overactive in individuals with chronic pain conditions (Bandeira et al., 2019). The *E-WAA* treatment led to a substantial decrease in the activation of FP and DLPFC, which provides a partial explanation of how *E-WAA* produces its analgesic effects. The results of the *E-WAA* treatment which revealed a notable reduction in the DLPFC partially corroborate the research which suggests the prefrontal cortex is involved in the experience of pain and is a component of the pain regulation system (Hibi et al., 2020). Moreover, it has been suggested that activity in the DLPFC is inversely associated with the severity of experienced pain and distress, suggesting that the DLPFC has a role in the cognitive control of pain (Bristot et al., 2020), and the modulation of this region can help suppress or amplify the transmission of pain signals through the subsequent inhibitory pathway. *E-WAA* therapy lessened the activity of the nociceptors, resulting in a decrease in the brain’s awareness of pain, relieving the brain tissue stimulation to some degree, and thus providing a measure of pain relief. Research has indicated that the decline in hemodynamic response could be connected to the production of endogenous opioids, and the contralateral hemisphere stress system will release a large amount of adrenocorticotropic hormone releasing factor, anti-peptides, and glutamate, leading to a lessening of the cortical hemodynamic response to pain. Subsequent to the application of *E-WAA* for a duration of 10 min, the activity levels of the two ROIs had not yet risen, leading to a dip in the release of dopamine from the brain’s hemispheric edges. Consequently, the body was still in a state of low dopamine energy, which was clinically demonstrated by a significant decrease in pleasure, motivation, and natural exuberance. Even the hemodynamic response had not been restored to its former state, suggesting that the participants were receiving an anesthetic effect from *E-WAA*, instead of adapting to the pain (Harada et al., 2005).

Although this research has some positive aspects, its drawbacks should not be ignored. The fact that the study only included males and the observed dissimilarities in pain tolerance between genders implies that more research and larger sample sizes are needed to ascertain the hemodynamic effects of *E-WAA* for pain relief, taking into consideration gender. In addition, we cannot overlook the impact of psychological cues and pain habituation related to pain on the outcomes, necessitating the use of more rigorous psychophysical methods to design future experiments.

## 5. Conclusion

This research employed a self-managed pre- and post-intervention evaluation with fNIRS to investigate the neural mechanisms of *E-WAA* in relieving pain in CSS sufferers. The findings of the study revealed that *E-WAA* could trigger bottom-up alleviation of pain, which was seemingly reflected in the alteration of HbO concentration in the PF, particularly demonstrated by the differences in the hemodynamic responses in the PF and DLPFC regions that are associated with pain. The data confirm that *E-WAA* is likely to have a broad range of clinical uses and that a quantitative assessment of HbO levels may be capable of elucidating the underlying mechanisms of *E-WAA*.

## Data availability statement

The original contributions presented in this study are included in the article/supplementary material, further inquiries can be directed to the corresponding authors.

## Ethics statement

The studies involving human participants were reviewed and approved by the Ethics Committee of ChiRCT. The patients/participants provided their written informed consent to participate in this study.

## Author contributions

JD and PS designed the study. JD collected and analyzed the data and manuscript editing. FF and HY supervised the writing of the manuscript. All authors approved the final manuscript.
